# Predicting acute kidney injury using urinary liver-type fatty-acid binding protein and serum N-terminal pro-B-type natriuretic peptide levels in patients treated at medical cardiac intensive care units

**DOI:** 10.1186/s13054-018-2120-z

**Published:** 2018-08-18

**Authors:** Hiroyuki Naruse, Junnichi Ishii, Hiroshi Takahashi, Fumihiko Kitagawa, Hideto Nishimura, Hideki Kawai, Takashi Muramatsu, Masahide Harada, Akira Yamada, Sadako Motoyama, Shigeru Matsui, Mutsuharu Hayashi, Masayoshi Sarai, Eiichi Watanabe, Hideo Izawa, Yukio Ozaki

**Affiliations:** 1 Department of Joint Research Laboratory of Clinical Medicine, Fujita Health University School of Medicine, 1-98 Kutsukake-cho, Dengakugakubo, Toyoake, 470-1192 Japan; 2 Division of Statistics, Fujita Health University School of Medicine, 1-98 Kutsukake-cho, Dengakugakubo, Toyoake, 470-1192 Japan; 3 Department of Cardiology, Fujita Health University School of Medicine, 1-98 Kutsukake-cho, Dengakugakubo, Toyoake, 470-1192 Japan; 4 Department of Cardiology, Banbuntane Houtokukai Hospital, 3-10 Otoubashi 3-cyome, Nakagawa-ku, Nagoya, 454-8509 Japan

**Keywords:** Liver-type fatty-acid binding protein, N-terminal pro-B-type natriuretic peptide, Acute kidney injury, Medical cardiac intensive care units

## Abstract

**Background:**

The early prediction of acute kidney injury (AKI) can facilitate timely intervention and prevent complications. We aimed to understand the predictive value of urinary liver-type fatty-acid binding protein (L-FABP) levels on admission to medical (non-surgical) cardiac intensive care units (CICUs) for AKI, both independently and in combination with serum N-terminal pro-B-type natriuretic peptide (NT-proBNP) levels.

**Methods:**

We prospectively investigated the predictive value of L-FABP and NT-proBNP for AKI in a large, heterogeneous cohort of patients treated in medical CICUs. Baseline urinary L-FABP and serum NT-proBNP were measured on admission. AKI was diagnosed according to the Kidney Disease: Improving Global Outcomes criteria. We studied 1273 patients (mean age, 68 years), among whom 46% had acute coronary syndromes, 38% had acute decompensated heart failure, 5% had arrhythmia, 3% had pulmonary hypertension, 2% had acute aortic syndrome, 2% had infective endocarditis, and 1% had Takotsubo cardiomyopathy.

**Results:**

Urinary L-FABP levels correlated with serum NT-proBNP levels (*r* = 0.17, *p* < 0.0001). AKI occurred in 224 patients (17.6%), including 48 patients with stage 2 or 3 disease. Patients who developed AKI had higher one-week and 6-month mortality than those who did not develop AKI (*p* = 0.0002 and *p* = 0.003, respectively). In the multivariate logistic analysis, both L-FABP (*p* < 0.0001) and NT-proBNP (*p* = 0.006) were independently associated with the development of AKI. Adding L-FABP and NT-proBNP to a baseline model that included established risk factors further improved reclassification (*p* < 0.001) and discrimination (*p* < 0.01) beyond that of the baseline model or any single biomarker individually.

**Conclusions:**

Urinary L-FABP and serum NT-proBNP levels on admission are independent predictors of AKI, and when used in combination, improve early prediction of AKI in patients hospitalized at medical CICUs.

## Background

Acute kidney injury (AKI) is a common clinical syndrome that affects critically ill patients and is strongly associated with increases in morbidity, mortality, and long-term loss of kidney function [1–3].

Serum creatinine is unable to identify early renal tubular injury before the decrease in glomerular filtration rate [4, 5], so an early and reliable biomarker of AKI is still needed to facilitate timely intervention and to prevent complications. However, it is also possible that a single biomarker may inadequately assess the risk of AKI because the syndrome is complex and multifactorial [4]. Combining different biomarkers in a single assessment could improve early prediction of AKI in the critically ill.

Liver-type fatty acid-binding protein (L-FABP), a marker of renal tubular injury, is an endogenous antioxidant protein spanning 14 kDa that is expressed in proximal tubular epithelial cells [6, 7]. Found in the cytoplasm of tubular cells, L-FABP is rapidly released into the tubular lumen in response to ischemia [8] or oxidative stress [8, 9]. To date, urinary L-FABP has been shown to be useful for the early prediction of AKI after surgery (particularly cardiac) [10, 11], sepsis [12], and emergency coronary angiography or percutaneous coronary intervention for acute coronary syndrome [13, 14]. However, there is limited information about the clinical utility of urinary L-FABP for predicting AKI in a heterogeneous cohort of patients treated in medical cardiac intensive care units (CICUs).

Another important and potentially relevant marker is N-terminal pro-B-type natriuretic peptide (NT-proBNP), a marker of hemodynamic stress [15]. High serum levels of NT-proBNP reflect hemodynamic deterioration, myocardial wall stress, myocardial ischemia, derangements in volume loading conditions, activation of the renin-angiotensin-aldosterone system and sympathetic nervous system, and renal dysfunction, all of which may lead to the development of AKI [16–20]. Several studies have also explored the association of NT-proBNP with the development of AKI after cardiac surgery [21] and coronary angiography or percutaneous coronary intervention [16, 22]. However, few studies have examined the clinical utility of NT-proBNP in medical CICUs.

In the present study, we prospectively investigated the predictive value of urinary L-FABP on admission, both independently and in combination with serum NT-proBNP, for predicting AKI in patients hospitalized to medical CICUs.

## Methods

### Study design

This prospective study was conducted at the Department of Cardiology, Fujita Health University School of Medicine (Toyoake, Japan). We enrolled patients hospitalized at medical CICUs at the Fujita Health University Hospital, 1435-bed tertiary-care academic medical center, from January 2016 to July 2017. Our medical CICU, a 10-bed unit, is staffed daily by eight cardiovascular care-trained attending physicians and three internal medicine residents. The nursing to patient ratio was 1:4. The study was approved by the Ethics Committee of Fujita Health University and conducted in accordance with the Declaration of Helsinki. All patients provided written informed consent.

Patients with cardiovascular disease requiring hospitalization as determined by the attending physician of the medical CICUs were eligible for enrollment. We obtained urinary and blood samples for baseline biomarker measurements on admission. The following patients were excluded: patients (1) under 18 years of age, (2) undergoing cardiac surgery, (3) experiencing trauma, (4) having end-stage renal disease (ESRD), (5) receiving percutaneous cardiopulmonary support before admission, (6) having active malignant disease being treated with chemotherapy or radiation, and (7) having autoimmune diseases. Independent physicians blinded to urinary L-FABP levels were free to select therapy as indicated. Clinical characteristics were obtained from patients’ medical records on enrollment, and patients were followed up for 6 months.

### Definitions and calculations

AKI was diagnosed according to the “Kidney Disease: Improving Global Outcomes” criteria, as an increase in serum creatinine by ≥ 0.3 mg/dL within 48 h or an increase in serum creatinine to ≥ 1.5 times the baseline within 1 week [23]. We used the lowest known serum creatinine value during the past 3 months as the baseline creatinine. For patients without known baseline, we used the lowest creatinine value within 7 days after admission at medical CICUs. The creatinine-based estimated glomerular filtration rate (eGFR) was calculated using the Modification of diet in renal disease study equation, as recommended by the Japan Chronic Kidney Disease Initiative [24]. ESRD was defined as receiving hemodialysis or having an eGFR < 15 mL/min/1.73 m^2^. Chronic kidney disease (CKD) was defined as an eGFR < 60 mL/min/1.73 m^2^ or a history of kidney transplantation.

Diabetes mellitus was defined as a history of or current diabetes mellitus and/or a fasting plasma glucose level ≥ 126 mg/dL, a hemoglobin A1c value ≥ 6.5%, or the presence of diabetic retinopathy. Hypertension was defined by systolic blood pressure ≥ 140 mmHg, diastolic blood pressure ≥ 90 mmHg, or a history of antihypertensive treatment. Dyslipidemia was defined as total cholesterol ≥ 220 mg/dL or a history of lipid-lowering therapy. For the smoking history, patients were defined as either current smokers or ex-smokers. We routinely performed two-dimensional echocardiography, from which the left ventricular ejection fraction (LVEF) was calculated using the modified Simpson method.

### Outcomes

The primary endpoint was the development of AKI. Urinary criteria were not used to diagnose AKI because of the inconsistent data recorded and the potential alterations in urine volume induced by medical therapy.

### Biomarker measurement

Urinary and blood samples were collected in nonheparinized tubes immediately after admission and centrifuged at 1000 g at 4 °C for 15 min, before being stored at − 80 °C until assayed. Urinary L-FABP was measured by a latex turbidimetric immunoassay, using a Norudia® L-FABP (Sekisui Medical CO., Ltd., Tokyo, Japan), which had a lower detection limit of 1.5 ng/mL. Urinary L-FAB*P* values below the lower detection limit of the assay were defined as 0.75 ng/mL. Serum NT-proBNP and high-sensitivity troponin T (hs-TnT) were measured using an electrochemiluminescence immunoassay and a Cobas® e601 system (Roche Diagnostics, Tokyo, Japan). Serum high-sensitivity C-reactive protein (hs-CRP) was measured using a latex-enhanced hsCRP immunoassay (N-Latex CRP II, Siemens Healthineers, Tokyo, Japan). Finally, the serum creatinine concentration was determined by an enzyme method, using the Liquitech® Creatinine PAP II (Roche Diagnostics, Tokyo, Japan) on admission, daily until day 3, and then on day 7.

### Data analysis

Statistical analyses were performed using StatFlex version 6 (Artech Co. Ltd. Osaka, Japan). Normally distributed variables are expressed as mean values ± standard deviations, and nonparametric data are presented as medians and interquartile ranges. Given the skewed distributions of the urinary L-FABP and serum NT-proBNP, hs-TnT, and hs-CRP data, analyses were performed after log-transformation to meet the criteria for use in normalized statistical approaches (after statistical confirmation). Intergroup differences were evaluated by one-way analysis of variance or the Kruskal–Wallis test for continuous variables and by the chi-square test for categorical variables. Intergroup differences in survival were examined using the Kaplan–Meier method and compared by the log-rank test. The odds ratios and 95% confidence intervals were calculated for each factor by logistic analysis: all baseline variables with *p* values <0.05 in the univariate analyses were entered into the multivariate logistic model to determine the independent predictors of AKI.

We calculated the C-index, net reclassification improvement (NRI), and integrated discrimination improvement (IDI) to assess whether the accuracy of predicting AKI would improve after adding L-FABP and NT-proBNP, or either factor in isolation, into a baseline model with established risk factors. The established risk factors were as follows: age, sex, hypertension, dyslipidemia, diabetes mellitus, smoking status, CKD, paroxysmal or persistent atrial fibrillation, acute decompensated heart failure, previous history of myocardial infarction, previous coronary revascularization, systolic blood pressure, heart rate, emergent coronary angiography, or percutaneous coronary intervention before admission, mechanical ventilation before admission, and intraaortic balloon pump before admission. The C-index was defined as the area under the receiver operating characteristic curves between individual predictive probabilities for AKI and the incidence of AKI, and it was compared with both the baseline model (established risk factors only) and the enriched models (baseline model plus L-FABP, NT-proBNP, or both) [25]. The NRI was a relative indicator of how many patients had an improvement in the predicted probability of AKI, whereas the IDI indicated the average improvement in the predicted probability of AKI after adding variables into the baseline model [26]. We considered *p* values <0.05 as statistically significant.

## Results

We enrolled 1273 patients aged 21–86 years and have summarized their demographics and clinical characteristics in Table 1. In total, 547 patients (43%) were diagnosed with CKD, with no case of kidney transplantation. A total of 588 patients (46%) were admitted because of acute coronary syndrome (241 patients had ST-segment elevation myocardial infarction, 292 had non-ST-segment elevation myocardial infarction, and 55 had unstable angina), 485 (38%) because of acute decompensated heart failure (244 patients with reduced ejection fraction (LVEF < 40%), 80 with mid-range ejection fraction (40% ≤ LVEF < 50%), and 161 with preserved ejection fraction (LVEF ≥ 50%)), 59 (5%) because of arrhythmia, 35 (3%) because of pulmonary hypertension, 27 (2%) because of acute aortic syndrome, 22 (2%) because of infective endocarditis, 15 (1%) because of Takotsubo cardiomyopathy, and 42 (3%), due to other causes (Table 2). Before admission, emergency coronary angiography or percutaneous coronary intervention was performed in 36%, mechanical ventilation was required for respiratory insufficiency in 2.1%, and intraaortic balloon pumps were needed for hemodynamic instability in 9% of patients.

**Table 1:** Baseline characteristics of study population according to AKI

	All	AKI	Non-AKI	*P* value
Number	1273	224	1049	
Age, years	68 ± 13	72 ± 11	67 ± 13	< 0.001
Male, *n* (%)	821 (64)	138 (62)	683 (65)	0.32
Hypertension, *n* (%)	811 (64)	169 (75)	642 (61)	< 0.001
Dyslipidemia, *n* (%)	575 (45)	88 (39)	487 (46)	0.05
Diabetes mellitus, *n* (%)	462 (36)	107 (48)	355 (34)	< 0.001
Current or ex-smoker, *n* (%)	361 (28)	59 (26)	302 (29)	0.46
Previous myocardial infarction, *n* (%)	228 (18)	41 (18)	187 (18)	0.87
Prior hospitalization for worsening heart failure, *n* (%)	242 (19)	45 (20)	197 (19)	0.65
Previous cerebral infarction, *n* (%)	181 (14)	41 (18)	140 (13)	0.05
Previous coronary revascularization, *n* (%)	231 (18)	41 (18)	190 (18)	0.95
Paroxysmal or persistent AF, *n* (%)	279 (22)	56 (25)	223 (21)	0.22
Acute decompensated heart failure, *n* (%)	485 (38)	118 (53)	367 (35)	< 0.001
Acute coronary syndrome, *n* (%)	588 (46)	77 (34)	511 (49)	< 0.001
Systolic blood pressure, mmHg	141 ± 31	144 ± 35	141 ± 30	0.14
Heart rate, beats per minutes	86 ± 26	89 ± 27	85 ± 25	0.04
Emergent CAG or PCI before admission, *n* (%)	458 (36)	68 (30)	390 (37)	0.05
Mechanical ventilation before admission, *n* (%)	27 (2.1)	10 (4.5)	17 (1.6)	0.007
IABP before admission, *n* (%)	115 (9)	39 (17)	76 (7)	< 0.001
White blood cell count, × 10^3^/μL	8.7 ± 3.7	9.8 ± 4.1	8.5 ± 3.5	< 0.001
Hemoglobin, g/dL	12.7 ± 2.3	12.0 ± 2.5	12.8 ± 2.3	< 0.001
eGFR, mL/min/1.73 m^2^	64.7 ± 23.1	51.3 ± 25.1	67.6 ± 21.6	< 0.001
Glucose, mg/dL	158 ± 69	182 ± 81	153 ± 66	< 0.001
hs-CRP, mg/L	2.49 (0.76–11.4)	5.52 (1.24–29.1)	2.13 (0.71–8.62)	< 0.001
NT-proBNP, pg/mL	1120 (230–4024)	2952 (1075–9329)	820 (194–3291)	< 0.001
hs-TnT, pg/mL	56 (16–443)	82 (26–661)	50 (15–408)	0.001
Urinary L-FABP, ng/mL	5.9 (2.4–18.0)	21.8 (6.3–65.9)	4.8 (2.0–12.9)	< 0.001
LVEF, %	47.5 ± 13.7	45.9 ± 14.5	47.9 ± 13.5	0.05
Treatment at enrollment, *n* (%)				
Antiplatelet drugs	425 (33)	83 (37)	342 (33)	0.20
Statins	395 (31)	67 (30)	328 (31)	0.69
RAAS inhibitors	518 (41)	103 (46)	415 (40)	0.08
Beta-blockers	329 (26)	66 (29)	263 (25)	0.17
Diuretics	338 (27)	77 (34)	261 (25)	0.004
Anticoagulant drugs	180 (14)	34 (15)	146 (14)	0.62

Data are expressed as number (%), mean ± standard deviation, or median (25th–75th percentile)

*AKI* acute kidney injury, *AF* atrial fibrillation, *CAG* coronary angiography, *PCI* percutaneous coronary intervention, *IABP* intraaortic balloon pump, *eGFR* creatinine-based estimated glomerular filtration rate, *hs-CRP* high-sensitivity C-reactive protein, *NT-proBNP* N-terminal pro-B-type natriuretic peptide, *hs-TnT* high-sensitivity cardiac troponin T, *L-FABP* liver-type fatty acid-binding protein, *LVEF* left ventricular ejection fraction, *RAAS* renin–angiotensin–aldosterone system

**Table 2:** Primary diagnosis

Diagnosis	Number (percentage)
Acute coronary syndrome, *n* (%)	588 (46)
STEMI, *n*	241
NSTEM, *n*	292
Unstable angina, *n*	55
Acute decompensated heart failure, *n* (%)	485 (38)
With reduced ejection fraction (LVEF < 40%), *n*	244
With mid-range ejection fraction (40% ≤ LVEF < 50%), *n*	80
With preserved ejection fraction (LVEF ≥ 50%), *n*	161
Arrhythmia, *n* (%)	59 (5)
Supraventricular tachycardia, *n*	8
Ventricular tachycardia, *n*	15
Sick sinus syndrome, *n*	17
Second-degree or third-degree atrioventricular block, *n*	19
Pulmonary hypertension, *n* (%)	35 (3)
Acute aortic syndrome, *n* (%)	27 (2)
Infective endocarditis, *n* (%)	22 (2)
Takotsubo cardiomyopathy, *n* (%)	15 (1)
Others, *n* (%)	42 (3)

Data are expressed as number (%)

*STEMI* ST-segment elevation myocardial infarction, *NSTEMI* non–ST-segment elevation myocardial infarction, *LVEF* left ventricular ejection fraction

Urinary L-FABP levels correlated with age (*r* = 0.06, *p* = 0.02), hemoglobin (*r* = − 0.06, *p* = 0.03), eGFR (*r* = − 0.28, *p* < 0.0001), hs-CRP (*r* = 0.21, *p* < 0.0001), NT-proBNP (*r* = 0.17, *p* < 0.0001), and hs-TnT levels (*r* = 0.26, *p* < 0.0001), and LVEF (*r* = − 0.07, *p* = 0.01). Serum NT-proBNPlevels correlated with age (*r* = 0.27, *p* < 0.0001), hemoglobin (*r* = − 0.31, *p* < 0.0001), eGFR (*r* = − 0.48, *p* < 0.0001), and hs-CRP levels (*r* = 0.44, *p* < 0.0001), and LVEF (*r* = − 0.46, *p* < 0.0001).

Of the 1273 patients, 224 (17.6%) developed AKI (48 had stage 2 or 3 disease). Compared with the non-AKI group, the patients in the AKI group were older; had higher heart rate; higher white blood cell count; higher levels of glucose, hs-CRP, NT-proBNP, hs-TnT, and L-FABP; and lower levels of hemoglobin, eGFR, and LVEF; more patients in the AKI group had the following: a history of intraaortic balloon pump or diuretic therapy; hypertension or diabetes mellitus; and acute decompensated heart failure (Table 1).

**Fig. 1:**
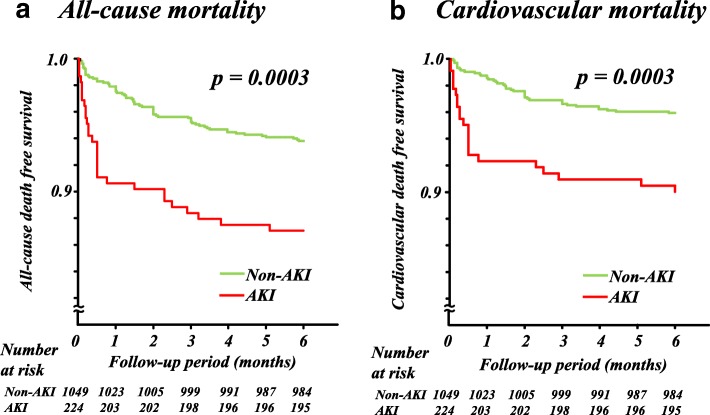
Kaplan–Meier analyses of all-cause (**a**) and cardiovascular (**b**) mortality by acute kidney injury (AKI)

The median length of CICU stay in the AKI group (4.0 (3.0–6.0) days) was longer than that in the non-AKI group (3.0 (2.0–4.0) days) (*p* < 0.001). During the 6-month follow-up period, there were 94 all-cause deaths (7.4%), of which 66 were cardiovascular related. During the first week after admission, 24 patients died, of which all deaths were related to cardiovascular issues. Patients with AKI had higher risk of 1-week mortality compared to those who did not (4.9% vs 1.2%, *p* = 0.0002). Patients with AKI had higher risks of all-cause death and cardiovascular death than patients without AKI (both, *p* = 0.0003) (Fig. 1).

**Fig. 2:**
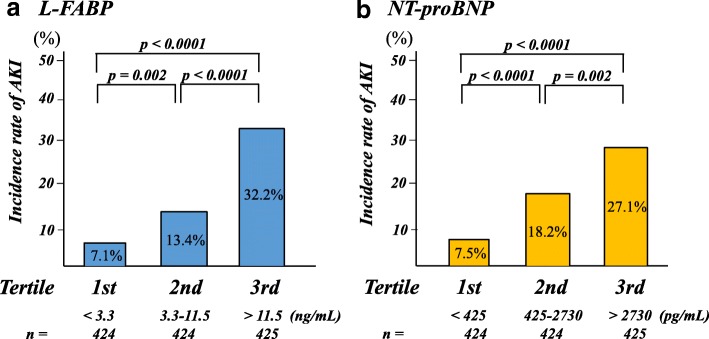
Incidence of acute kidney injury (AKI) by liver-type fatty-acid binding protein (L-FABP) (**a**) and N-terminal pro-B-type natriuretic peptide (NT-proBNP) (**b**) tertiles

**Fig. 3:**
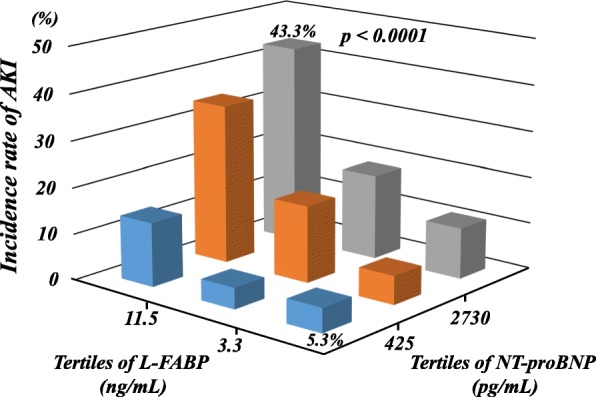
Incidence of acute kidney injury (AKI) when combining L-FABP liver-type fatty-acid binding protein (L-FABP) and N-terminal pro-B-type natriuretic peptide (NT-proBNP) tertiles

Patients were divided into tertiles according to their urinary L-FABP and serum NT-proBNP levels. In the multivariate logistic analyses, both L-FABP and NT-proBNP were independent predictors of AKI when assessed as either continuous variables (*p* < 0.0001 and *p* = 0.006, respectively) or variables categorized by tertile (*p* < 0.0001 and *p* = 0.009, respectively, when comparing the third tertile with the first tertile) (Table 3). Intraaortic balloon pump use and the eGFR also remained significantly associated with AKI. Moreover, Fig. 2 shows that the incidence of AKI gradually increased as the L-FABP and NT-proBNP tertile increased. As shown in Fig. 3, patients in whom both markers were in the highest tertile had the highest risk of AKI, whereas those who had both biomarkers in the lowest tertile had the lowest risk of AKI (*p* < 0.0001).

**Table 3:** Multivariate logistic analyses of predictors of AKI

Variables	Multivariate model 1		Multivariate model 2	
	OR (95% CI)	*P* value	OR (95% CI)	*P* value
Age (per 10 years increment)	1.15 (0.98–1.37)	0.09	1.16 (0.98–1.37)	0.08
Hypertension	1.42 (0.97–2.10)	0.07	1.33 (0.91–1.94)	0.14
Diabetes mellitus	1.31 (0.90–1.91)	0.16	1.26 (0.87–1.83)	0.22
Acute decompensated heart failure	1.26 (0.81–1.96)	0.30	1.47 (0.96–2.25)	0.08
Mechanical ventilation before admission	1.24 (0.47–3.27)	0.67	1.30 (0.51–3.30)	0.58
IABP before admission	2.49 (1.41–4.42)	0.002	2.48 (1.40–4.37)	0.002
Heart rate (per 10 beats per minute increment)	1.01 (0.94–1.08)	0.82	1.02 (0.95–1.09)	0.54
White blood cell count (per ×10^3^/μL increment)	1.04 (0.99–1.09)	0.17	1.04 (0.99–1.09)	0.11
Hemoglobin (g/dL) (per 1 g/dL increment)	0.94 (0.87–1.02)	0.14	0.94 (0.87–1.02)	0.13
eGFR (per 10 mL/min/1.73m^2^ increment)	0.91 (0.83–0.99)	0.03	0.85 (0.78–0.92)	< 0.001
Glucose (per 10 mg/dL increment)	1.02 (0.99–1.04)	0.20	1.02 (1.00–1.05)	0.10
hs-CRP (per 10-fold increment)	1.06 (0.85–1.34)	0.60	1.15 (0.92–1.44)	0.21
NT-proBNP (per 10-fold increment)	1.58 (1.14–2.19)	0.006		
Tertile of NT-proBNP (pg/mL)				
First (< 425)			1.0	
Second (425–2730)			1.79 (0.98–3.25)	0.06
Third (> 2730)			1.97 (1.19–3.26)	0.009
hs-TnT (per 10-fold increment)	0.96 (0.78–1.18)	0.67	1.02 (0.83–1.24)	0.88
Urinary L-FABP (per 10-fold increment)	2.66 (2.03–3.48)	< 0.001		
Tertile of Urinary L-FABP (ng/mL)				
First (< 3.3)			1.0	
Second (3.3–11.5)			1.42 (0.87–2.30)	0.16
Third (> 11.5)			3.40 (2.13–5.44)	< 0.001
LVEF (per 10% increment)	1.12 (0.98–1.29)	0.11	1.11 (0.96–1.27)	0.15
Diuretics	0.98 (0.68–1.49)	0.98	0.93 (0.64–1.37)	0.71

Multivariate model adjusted for all baseline variables with *p* < 0.05 on univariate analysis. NT-proBNP and L-FABP levels were assessed as either continuous variables (model 1) or variables categorized into tertiles (model 2)

*AKI* acute kidney injury, *OR* odds ratio, *CI* confidence interval, *IABP* intraaortic balloon pump, *eGFR* creatinine-based estimated glomerular filtration rate, *hs-CRP* high-sensitivity C-reactive protein, *NT-proBNP* N-terminal pro-B-type natriuretic peptide, *hs-TnT* high-sensitivity cardiac troponin T, *L-FABP* liver-type fatty acid-binding protein, *LVEF* left ventricular ejection fraction

Finally, we looked at the effect of adding L-FABP, NT-proBNP, or both to a baseline model of established risk factors. As shown in Table 4, the significant increase in the C-index indicated that adding L-FABP improved the prediction of AKI beyond that of the baseline model alone. Furthermore, adding both L-FABP and NT-proBNP significantly improved the reclassification of patients beyond that of adding any single biomarker and beyond that of the baseline model alone (all *p* < 0.001); the IDI improved similarly after adding both L-FABP and NT-proBNP (all *p* < 0.01).

**Table 4:** Discrimination and reclassification of the combination of L-FABP and NT-proBNP for AKI

	C-index	*P* value	NRI	*P* value	IDI	*P* value
Established risk factor model	0.741	Ref.		Ref.		Ref.
Established risk factor model + NT-proBNP	0.762	0.38	0.316	<0.001	0.018	<0.001
Established risk factor model + L-FABP	0.794	0.03	0.561	<0.001	0.092	<0.001
Established risk factor model + NT-proBNP + L-FABP	0.803	0.01	0.606	<0.001	0.101	<0.001
Established risk factor model + NT-proBNP + L-FABP vs	0.041*	0.08	0.513	<0.001	0.083	<0.001
Established risk factor model + NT-proBNP						
Established risk factor model + NT-proBNP + L-FABP vs	0.009*	0.70	0.256	<0.001	0.009	0.006
Established risk factor model + L-FABP						

Established risk factors included age, sex, hypertension, dyslipidemia, diabetes mellitus, smoking status, chronic kidney disease, atrial fibrillation, acute decompensated heart failure, previous myocardial infarction, previous coronary revascularization, systolic blood pressure, heart rate, emergent coronary angiography or percutaneous coronary intervention before admission, mechanical ventilation before admission, intraaortic balloon pump before admission

*L-FABP* liver-type fatty acid-binding protein, *NT-proBNP* N-terminal pro-B-type natriuretic peptide, *AKI* acute kidney injury, *NRI* net reclassification improvement, *IDI* integrated discrimination improvement, *Ref.* reference

*Estimated differences between two groups

## Discussion

The main findings of this study were as follows. First, admission levels of both urinary L-FABP and serum NT-proBNP were independent predictors of developing AKI in patients treated in medical CICUs. Second, patients with L-FABP and NT-proBNP in the upper tertiles were most strongly associated with an increased risk of AKI. Third, combining L-FABP and NT-proBNP improved the predictive value for AKI beyond that achieved with any single biomarker or baseline model alone, as demonstrated by the NRI and IDI. Fourth and finally, patients who developed AKI had a higher risk of death at 1 week and at 6 months than patients who did not develop AKI. These findings indicate that assessing both urinary L-FABP and serum NT-proBNP levels on admission may help with the early risk stratification of AKI in patients admitted to medical CICUs.

Medical CICUs have evolved from their origins of focusing exclusively on patients with acute coronary syndromes and now provide comprehensive critical care to patients with a range of acute cardiovascular illnesses and complex comorbidities [27, 28]. These include patients with diabetes mellitus, hypertension, dyslipidemia, and CKD, and those who use therapeutic devices. Recent prospective studies have shown that, as with patients treated in surgical intensive care units, AKI is commonly and is strongly associated with increased mortality [27]. However, the utility of urinary L-FABP for predicting AKI has not been fully evaluated in medical CICU settings, and there was a need to confirm the reliability and generalizability of L-FABP in heterogeneous populations before its clinical use could be advocated. Thus, we, for the first time, demonstrated that the combined use of urinary L-FABP and serum NT-proBNP, which are individually independent predictors of AKI, could improve the early prediction of AKI in a large (*n* = 1273), heterogeneous cohort of patients treated at medical CICUs not including cardiac surgery cases.

Type 1 cardiorenal syndrome reflects an abrupt worsening of cardiac function leading to AKI [29]. The combination of urinary L-FABP, a marker of renal tubular injury, and NT-proBNP, a marker of hemodynamic stress, to assess for the development of the cardiorenal syndrome is conceptually attractive. The data presented herein not only support both of these as independent predictors of AKI but also show that combining L-FABP and NT-proBNP can improve the risk reclassification and discrimination for AKI among medical patients in the CICU. Indeed, patients whose results for both markers were in the highest tertile were approximately eightfold more likely to develop AKI than those whose results for both markers were in the lowest tertile. It is crucial that these biomarkers benefit from being readily measurable, easily accessible, relatively inexpensive, and reproducible with high sensitivity and specificity. Following confirmation of our data in other independent cohorts, this combination approach to screening could be included in an algorithm for predicting AKI on admission to a CICU.

In the present study, urinary L-FABP was weakly (*r* = 0.17), although statistically significantly, correlated with NT-proBNP. This weak correlation suggests that they may target two different pathophysiological dysregulations of AKI: renal tubular injury and hemodynamic stress. Also, the coefficient for correlation between urinary L-FABP and age and between urinary L-FABP hemoglobin was less than 0.2, which was consistent with the results of previous studies [30, 31].

Recently, the predictive value of combining urinary L-FABP with other urinary AKI biomarkers, such as neutrophil gelatinase-associated lipocalin (NGAL) [32, 33], interleukin-18 [34], or kidney injury molecule-1 [11], for the development of AKI after cardiac surgery have been studied. Especially, a combination of urinary L-FABP and NGAL may be the most promising strategy for predicting AKI. With respect to the plasma NGAL evaluation along with B-type natriuretic peptide (BNP) in acutely decompensated heart failure, the GALLANT trial showed prognostic values for plasma NGAL, alone and in combination with BNP in acutely decompensated heart failure [35]. These findings lead us to speculate that a biomarker panel consisting of L-FABP, BNP or NT-proBNP, and urinary or plasma NGAL may improve the early prediction of AKI in patients admitted to medical CICUs. Further investigation is desired to confirm the usefulness of this approach.

It should be noted that we measured urinary L-FABP only at the point of admission to the CICU. The decision for this was based on reports of whether this is the best time for predicting AKI in patients with acute decompensated heart failure [36] or whether it is after cardiac surgery [37]. Thus, although we showed that admission measurements of urinary L-FABP may help physicians predict AKI, we do not know whether L-FABP, NT-proBNP, or a combination of both could also be used for monitoring markers. Indeed, we do not know whether improvements in these biomarkers would have affected the study outcomes.

The treatments in this study were also not randomized or controlled, making it difficult to evaluate the effects of treatments on the progression of AKI. For example, AKI was significantly associated with the frequent use of diuretics, whereas some patients underwent emergency coronary angiography or percutaneous coronary intervention and intraaortic balloon pump insertion before samples were obtained. These differences in treatment strategy may have had potentially significant confounding effects on the study results. However, even when we entered these factors into our multivariate logistic analyses, both L-FABP and NT-proBNP remained valid, significant, independent predictors of AKI. Thus, we believe that these did not contribute to significant confounding.

Observational studies suggest an association between AKI and the subsequent development of CKD and ESRD, even following apparent renal recovery [3, 38]. Thus, it is interesting to see if urinary L-FABP could predict CKD and ESRD. However, we could not evaluate this issue because of the study design focused on the early prediction of AKI. Nevertheless, when we retrospectively collected serum creatinine data between 3 and 6 months after discharge in 926 patients (73%) from the study group, the progression to ESRD occurred in 29 patients, including 7 patients who received hemodialysis. The incidence rate for the progression to ESRD in patients with AKI was significantly higher than in patients without AKI (15.6% vs 0.5%, *p* < 0.0001). Patients in the third tertile of L-FABP had the highest risk of the progression to ESRD (*p* < 0.0001, when compared with patients in both the first or second tertile; 8.8% vs 0.6% or 0.7%), speculating that higher levels of urinary L-FABP may be associated with the development of ESRD. A larger prospective study is needed to clarify this issue.

There are a few other limitations in the present study. First, it was conducted at a single institution. Second, we evaluated urinary L-FABP as the absolute concentration, but did not use urinary creatinine correction, because the urinary creatinine excretion rate may change over time under non-steady-state conditions [39]. Nevertheless, when we analyzed L-FABP using urinary creatinine correction, the results were comparable with those obtained without creatinine correction (data not shown). Further investigation is necessary to determine the optimal normalization for urinary L-FABP. Third, AKI was only defined using the elevation of serum creatinine because of the inconsistent data recorded and the potential alterations in urine volume induced by medical therapy. This may lead to the neglect of a part of the renal insult, which may be determined by urine output. Finally, the incidence of AKI in this study was 17.6%, which was lower than that reported in two recent studies (28.7% and 31.6%) [40, 41]. This difference may be attributable to differences in the severity of patients’ disease, with higher frequencies of mechanical ventilation and higher values for NT-proBNP in the previous research.

## Conclusion

Combining measurements for two independent predictors of AKI, namely, urinary L-FABP and serum NT-proBNP, when patients are admitted to a CICU, improves the early prediction of AKI beyond that achieved when using either predictor in isolation.

## Abbreviations

AKI: Acute kidney injury
BNP: B-type natriuretic peptide
CICUs: Cardiac intensive care units
CKD: Chronic kidney disease
eGFR: Creatinine-based estimated glomerular filtration rate
ESRD: End-stage renal disease
hs-CRP: High-sensitivity C-reactive protein
hs-TnT: High-sensitivity troponin T
IDI: Integrated discrimination improvement
L-FABP: Liver-type fatty acid-binding protein
LVEF: Left ventricular ejection fraction
NGAL: Neutrophil gelatinase-associated lipocalin
NRI: Net reclassification improvement
NSTEMI: Non–ST-segment elevation myocardial infarction
NT-proBNP: N-terminal pro-B-type natriuretic peptide
STEMI: ST-segment elevation myocardial infarction
